# The food crisis in the context of climate change and sustainable development goals

**DOI:** 10.17843/rpmesp.2023.404.13553

**Published:** 2023-12-18

**Authors:** J. Jaime Miranda, Carol Zavaleta-Cortijo

**Affiliations:** 1 Sydney School of Public Health, Faculty of Medicine and Health, University of Sydney, Sídney, Australia. University of Sydney Sydney School of Public Health Faculty of Medicine and Health University of Sydney Sídney Australia; 2 CRONICAS Center of Excellence in Chronic Diseases, Universidad Peruana Cayetano Heredia, Lima, Peru. Universidad Peruana Cayetano Heredia CRONICAS Center of Excellence in Chronic Diseases Universidad Peruana Cayetano Heredia Lima Peru; 3 Unit for Intercultural Citizenship and Indigenous Health (UCISI), School of Public Health and Administration, Universidad Peruana Cayetano Heredia, Lima, Peru. Universidad Peruana Cayetano Heredia Unit for Intercultural Citizenship and Indigenous Health (UCISI) School of Public Health and Administration Universidad Peruana Cayetano Heredia Lima Peru

The 2030 Agenda is a comprehensive, progressive and innovative agenda that responds to the many challenges the world faces today and will face in the future. It aims to integrate the social, environmental and economic dimensions of sustainable development. The 2030 Agenda includes the 17 Sustainable Development Goals (SDGs), which are a roadmap for development efforts towards 2030 and beyond. In other words, the 2030 Agenda and the SDGs form a plan of action focused on people, planet and prosperity.

The SDGs were established in 2015, with a 15-year deadline. This year, 2023, is halfway to that deadline and progress towards meeting the SDGs is not encouraging ^1^^,^^2^. The recent Global Sustainable Development Report points out some key messages to motivate world leaders to get back on track towards 2030 ^1^. First, there is a need to implement desirable and positive transformations to accelerate progress toward 2030, and for this, knowing and understanding the science of transformations is essential. Then, progress focused on isolated objectives is not efficient, so working on more than one objective simultaneously opens a window of opportunity. This is why we should address, in a strategic and transparent manner, the connections and interrelationships between various SDGs in order to maximize and enhance positive interrelationships, as well as anticipate and minimize negative interrelationships. Finally, some transformations should be prioritized and positive interrelationships should be mapped in addition to anticipating negative ones, which requires local capacity and talent; therefore, it is necessary to foster capacities that not only understand the various local contexts but also have the capacity to make decisions.

The food crisis and climate change are not unfamiliar to Peru. The food crisis is the focus of SDG 2, zero hunger, while climate change is central to SDG 13, Climate Action. Food plays an essential role in human survival and climate change, and therefore has a direct impact on each and every one of the other SDGs: End Poverty (SDG 1), Health and Wellbeing (SDG 3), Education and Quality (SDG 4), Gender Equality (SDG 5), Clean Water and Sanitation (SDG 6), Affordable and Clean Energy (SDG 7), Decent Work and Economic Growth (SDG 8), Industry Innovation and Infrastructure (SDG 9), Reducing Inequalities (SDG 10), Sustainable Cities and Communities (SDG 11), Responsible Production and Consumption (SDG 12), Underwater Life (SDG 14), Life of Terrestrial Ecosystems (SDG 15), Peace, Justice and Strong Institutions (SDG 16), and Partnerships to Achieve the Goals (SDG 17). So, if the goal is to engage in transformations needed to accelerate progress, the question arises, how do we outline, anticipate and prioritize activities that will maximize progress in confronting the food crisis and climate change alongside the other SDGs? Part of this exercise requires a focus on synergies, as strategies that simultaneously target improvements in more than one SDG must be prioritized.

In Peru we have seen sustained efforts from the health and public health sectors -perhaps somewhat isolated- to address some indicators linked to healthy eating, from programs to combat chronic malnutrition and anemia, to general policies such as the Healthy Eating Law. Peru, a country so diverse in population and ecosystems, is also highly vulnerable to climate change. This vulnerability, and the vision of a prosperous planet, confronts us with new challenges on a personal and collective level at different levels of government agency structures and community spaces, including the private sector as well as the public sector. From how we consume our food and reduce organic and inorganic waste, to what we do to protect the most vulnerable populations.

Achieving the vision of a prosperous planet and healthy societies requires significant transformations in our daily behavior and even in the formulation of public policies; these transformations also bring multiple opportunities with them. For example, cleaner electric vehicle technology has been steadily gaining ground in several countries. In Peru, access to a unique diversity of food products, coupled with the social and cultural value associated with food and nutrition, strategically positions us to face the challenge of generating positive transformations that promote a sustainable planet and reflect the priorities in our local context.

Climate change gives us the opportunity to build a sustainable, nutritious and equitable food system, aimed at closing nutritional gaps. For example, for the first time, we recognize and seek to address the “hidden costs” of food systems, which are those costs that have negative impacts on both the environment and health ^3^. Greenhouse gas emissions, water pollution, loss of biodiversity, and dietary patterns that lead to the development of chronic diseases are some of the hidden ecological, social, and health costs that food systems transformation seeks to change.

On the other hand, climate change has a direct impact on nutrition, which is evident through several mechanisms. One of them is the increase in food insecurity, by reducing food production and access to food, and also due to significant changes in dietary diversity, which mainly affect middle and low-income populations. The intersection between climate, food, nutrition and health forces us to create strategies to adapt to climate threats, which must include multisectoral and comprehensive policies and actions. This implies that the transformation must take a broader view and not focus solely on food production and quality. Water and sanitation services, social protection strategies, as well as recognition and respect for different food patterns, usually related to cultural diversity, must also be addressed ^4^.

Subsistence family farming dominates food production in countries such as Peru ^(^^5^. Food production for self-consumption is favored in the highlands and jungle regions, while in the coastal region it is oriented towards the market, partly due to the proximity to large urban centers with greater population and volume of demand. At the same time, the type of food produced or generated is different in these three regions, which also has repercussions on dietary patterns and relationships with food. Transformation implies a change in values, as well as learning from past events, particularly how to respond to extreme weather events in order to take advantage of the characteristics that already exist in current food systems. The transformation that the country needs must promote the revaluation of “knowing, eating and drinking” and of the primary actors in the production and generation of food. For example, the preference for fresh foods resistant to droughts and floods, the ancestral knowledge of our people about crops and wild species, represents a food system that in addition to its nutritional value, has collective social value, because it includes valuable foods during and after extreme weather events.

The Peruvian context, in a chronic crisis of poor nutritional indicators coupled with notorious climate vulnerability, calls for actions based on the efficient and strategic use of available resources, including human talent. If we configure these challenges together with the recommendations of the Global Sustainable Development Report 2023, we will find very concrete opportunities. As previously stated, strategies should be focused on accelerating positive transformations, especially those transformations that are useful and relevant to the multiple contexts of our country. Then, the interrelationships between several SDGs must be considered beyond SDGs 2 and 13, which means leaving behind the mentality of working in silos. Finally, it is important to foster local capacity and talent to have agency and decision-making capacity in the transformations that are essential to lead to a prosperous planet with concrete local repercussions, recognizing local voices, priorities and knowledge. Particularly, the Global Sustainable Development Report 2023 states that “transformations are possible and inevitable”, and calls on all leaders of the planet to establish, by July 2024, transformational platforms to accelerate action on the SDGs ^1^. It is possible, transformations are inevitable.
