# Supplementary material for: The food crisis in the context of climate change and sustainable development goals
Source: Rev Peru Med Exp Salud Publica. 2023 Dec 18;40(4):392–4. doi: 10.17843/rpmesp.2023.404.13553 (PMC11138824; doi:10.17843/rpmesp.2023.404.13553)
Supplement: Translation

# Translation: La crisis alimentaria en el contexto del cambio climático y los objetivos de desarrollo sostenible

**Authors:** J. Jaime Miranda, Carol Zavaleta-Cortijo

**Affiliations:** 1 Sydney School of Public Health, Faculty of Medicine and Health, University of Sydney, Sidney, Australia.; 2 CRONICAS Centro de Excelencia en Enfermedades Crónicas, Universidad Peruana Cayetano Heredia, Lima, Perú.; 3 Unidad de Ciudadanía Intercultural y Salud Indígena (UCISI), Facultad de Salud Pública y Administración, Universidad Peruana Cayetano Heredia, Lima, Perú.

La Agenda 2030 es una agenda integral, progresista e innovadora que responde a los numerosos desafíos que el mundo enfrenta hoy y enfrentará en el futuro. Tiene como objetivo integrar las dimensiones social, ambiental y económica del desarrollo sostenible. La Agenda 2030 incluye a los 17 Objetivos de Desarrollo Sostenible (ODS), los cuales son una hoja de ruta para los esfuerzos de desarrollo con miras al 2030 y más allá. En otras palabras, la Agenda 2030 y los ODS configuran un plan de acción centrado en las personas, el planeta y la prosperidad.

Los ODS fueron establecidos en el año 2015, con un plazo de 15 años. Este año, 2023, se encuentra a la mitad de dicho plazo y el progreso hacia el cumplimiento de los ODS no es alentador ^1^^,^^2^. El reciente Reporte Global de Desarrollo Sostenible señala algunos mensajes clave para motivar a los líderes mundiales a retomar el rumbo hacia el 2030 ^1^. Primero, existe la necesidad de implementar transformaciones deseables y positivas para acelerar el progreso hacia el 2030, y para ello, conocer y entender la ciencia de las transformaciones es esencial. Segundo, el avance centrado en objetivos aislados no es eficiente, por lo que trabajar en más de un objetivo de manera simultánea abre una ventana de oportunidades. Es por ello que se invita a abordar, de manera estratégica y transparente, las conexiones e interrelaciones entre varios ODS con el fin de maximizar y potenciar las interrelaciones positivas, así como también anticipar y minimizar las interrelaciones negativas. Tercero, se debe decidir qué transformaciones deben priorizarse y cómo se debe mapear las interrelaciones positivas además de anticipar las negativas, lo cual requiere de capacidad y talento local; para esto es necesario fomentar capacidades que no solamente conozcan los diversos contextos locales sino también que cuenten con la capacidad de tomar decisiones.

La crisis alimentaria y el cambio climático no son ajenos al Perú. La crisis alimentaria es el foco del ODS 2, hambre cero, mientras que el cambio climático es central en el ODS 13, Acción por el Clima. La alimentación tiene un rol esencial en la sobrevivencia humana y en el cambio climático, por lo tanto impacta directamente en todos y cada uno de los demás ODS: Fin de la Pobreza (ODS 1), Salud y Bienestar (ODS 3), Educación y Calidad (ODS 4), Igualdad de Género (ODS 5), Agua Limpia y Saneamiento (ODS 6), Energía asequible y no Contaminante (ODS 7), Trabajo Decente y Crecimiento Económico (ODS 8), Industria Innovación e Infraestructura (ODS 9), Reducción de las Desigualdades (ODS 10), Ciudades y Comunidades Sostenibles (ODS 11), Producción y consumo responsable (ODS 12), Vida Submarina (ODS 14), Vida de Ecosistemas Terrestres (ODS 15), Paz, Justicia e Instituciones Sólidas (ODS 16), y Alianzas para Lograr los Objetivos (ODS 17). Entonces, si el objetivo es embarcarse en transformaciones necesarias para acelerar el progreso, se plantea la siguiente pregunta, ¿cómo delinear, anticipar y priorizar actividades que maximicen el progreso para confrontar la crisis alimentaria y el cambio climático junto a los otros ODS? Parte de este ejercicio requiere enfocarse en sinergias, dado que se deben priorizar estrategias que apunten a mejoras, de manera simultánea, en más de un ODS.

En Perú hemos visto esfuerzos sostenidos desde los sectores de salud y salud pública -quizás de alguna manera aislados- por abordar algunos indicadores vinculados a la alimentación saludable, desde programas para combatir la desnutrición crónica y la anemia, hasta políticas generales como la Ley de Alimentación Saludable. El Perú, un país tan diverso en poblaciones y ecosistemas, es también altamente vulnerable al cambio climático. Esta vulnerabilidad, y la visión de un planeta próspero, nos enfrenta a nuevos retos a nivel personal y colectivo en los distintos niveles de estructuras de organismos de gobierno y espacios comunitarios, incluyendo al sector privado además del público. Desde cómo consumimos nuestros alimentos y reducimos los desperdicios orgánicos e inorgánicos, hasta qué hacemos para proteger a las poblaciones más vulnerables.

Alcanzar la visión de un planeta próspero y sociedades saludables requiere de transformaciones notorias con respecto a nuestro comportamiento cotidiano e incluso la formulación de políticas públicas; estas transformaciones también conllevan múltiples oportunidades. Por ejemplo, la tecnología para vehículos eléctricos menos contaminantes viene estableciéndose a paso firme en varios países. En nuestro país, el acceso a una diversidad única de productos alimentarios sumado al valor social y cultural asociado a la comida y alimentación, nos posiciona, de manera estratégica, frente al reto de generar transformaciones positivas que promuevan un planeta sostenible y que reflejen las prioridades en nuestro contexto local.

El cambio climático nos da la oportunidad de construir un sistema alimentario sostenible, nutritivo y equitativo, con miras a cerrar las brechas nutricionales. Por ejemplo, por primera vez se reconoce y se busca abordar los “costos ocultos” de los sistemas alimentarios, que son aquellos costos que tienen repercusiones negativas tanto en el medio ambiente como en la salud ^(^^3^. La emisión de gases de efecto invernadero, la contaminación del agua, la pérdida de la biodiversidad y los patrones de dieta que llevan al desarrollo de enfermedades crónicas son algunos de los costos ocultos, ecológicos, sociales y de la salud, que la transformación de los sistemas alimentarios busca cambiar.

Por otro lado, el cambio climático tiene un impacto directo en la nutrición, el cual se hace evidente a través de varios mecanismos. Uno de ellos es el incremento de la inseguridad alimentaria, al reducir la producción y el acceso a los alimentos, y también debido a los cambios significativos en la diversidad de la dieta, que afectan principalmente a poblaciones de medianos y bajos recursos económicos. La intersección entre clima, alimentos, nutrición y salud nos obliga a crear estrategias para adaptarnos a los peligros climáticos, las cuales deben incluir políticas y acciones multisectoriales e integrales. Esto implica que la transformación debe tener una mirada más amplia y no enfocada únicamente, en la producción y calidad de alimentos. Se requiere abordar también los servicios de agua y saneamiento, las estrategias de protección social, así como el reconocimiento y respeto de diferentes patrones alimentarios, usualmente relacionados a la diversidad cultural ^4^.

En países como el Perú, la agricultura familiar de subsistencia domina la producción de alimentos ^5^. En las regiones de la sierra y la selva se favorece la producción de alimentos para autoconsumo; mientras que en la región de la costa se orienta hacia el mercado, en parte debido a la cercanía a los grandes centros urbanos con mayor población y volumen de demanda. Al mismo tiempo el tipo de alimentos que se produce o se genera es diferente en estas tres regiones, lo cual también repercute en diferentes patrones de dieta y relaciones con los alimentos. La transformación implica un cambio de valores, así como también aprender de eventos pasados, particularmente, de la forma de respuesta ante eventos climáticos extremos con el objetivo de tomar ventaja de las características que ya existen en los sistemas de alimentación. La transformación que el país necesita debe promover la revalorización del “saber, comer y beber” y de los actores primarios en la producción y generación del alimento. Por ejemplo, la preferencia por alimentos frescos resistentes a las sequías e inundaciones, el conocimiento ancestral de nuestros pueblos sobre cultivos y especies silvestres, representa un sistema de alimentación que además del valor nutricional, tiene valor social colectivo, debido a que incluye alimentos valiosos durante y después de eventos climáticos extremos.

El contexto peruano, situado en una crisis crónica de pobres indicadores nutricionales sumado a una notoria vulnerabilidad climática, llama a tomar acciones basadas en el uso eficiente y estratégico de los recursos disponibles, incluyendo el talento humano. Si configuramos estos retos junto con las recomendaciones del Reporte Global de Desarrollo Sostenible 2023 encontraremos oportunidades muy concretas. Según lo señalado previamente, las estrategias deben estar enfocadas en acelerar transformaciones positivas, sobre todo aquellas transformaciones que son útiles y relevantes para los múltiples contextos de nuestro país. Luego, se deben considerar las interrelaciones entre varios ODS, más allá de los ODS 2 y 13, lo que significa dejar atrás la mentalidad de trabajo en silos. Finalmente, es importante fomentar la capacidad y talento local para tener agencia y capacidad de decisión en las transformaciones que son esenciales para conllevar a un planeta próspero con repercusiones locales concretas, reconociendo las voces, prioridades y conocimiento local. De manera específica, el Reporte Global de Desarrollo Sostenible 2023 señala que las “transformaciones son posibles e inevitables”, y hace un llamado a todos los líderes del planeta a establecer, a julio del 2024, plataformas de transformación para acelerar acciones frente a los ODS ^1^. Es posible, las transformaciones son inevitables.
